# Acute abdominal compartment syndrome complicating a colonoscopic perforation: a case report

**DOI:** 10.1186/1752-1947-6-51

**Published:** 2012-02-06

**Authors:** Amine Souadka, Raouf Mohsine, Lahsen Ifrine, Abdelkader Belkouchi, Hadj Omar El Malki

**Affiliations:** 1 Surgical Department A, Ibn Sina Hospital, Rabat, Morocco; 2 Medical Center of Clinical Trials and Epidemiological Study (CRECET), Medical School, University Mohammed Vth Souissi, Rabat, Morocco; 3 Biostatical, Clinical Research and Epidemiological Laboratory (LBRCE), Medical School, University Mohammed Vth Souissi, Rabat, Morocco

## Abstract

**Introduction:**

A perforation occurring during colonoscopy is an extremely rare complication that may be difficult to diagnose. It can be responsible for acute abdominal compartment syndrome, a potentially lethal complex pathological state in which an acute increase in intra-abdominal pressure may provoke the failure of several organ systems.

**Case presentation:**

We report a case of acute abdominal compartment syndrome after perforation of the bowel during a colonoscopy in a 60-year-old North African man with rectal cancer, resulting in respiratory distress, cyanosis and cardiac arrest. Our patient was treated by needle decompression after the failure of cardiopulmonary resuscitation. An emergency laparotomy with anterior resection, including the perforated sigmoid colon, was then performed followed by immediate anastomosis. Our patient remains alive and free of disease three years later.

**Conclusion:**

Acute abdominal compartment syndrome is a rare disease that may occasionally occur after a colonoscopic perforation. It should be kept in mind during colonoscopy, especially considering its simple salvage treatment.

## Introduction

Colonic perforation due to colonoscopy rarely occurs but still remains a major complication with a high rate of morbidity and mortality and often needs surgical management [1]. The frequency of perforation is estimated to be 0.03% to 0.9% for diagnostic colonoscopy and 0.15% to 2% for therapeutic colonoscopy [2-4]. In diagnostic procedures, perforations often result from the pressure on the colonic wall, especially in ones noted to be a 'difficult procedure' by the endoscopist [5]. The diagnosis is often suspected after the visualization of extra-intestinal tissue. It is well established that therapeutic colonoscopies are associated with a higher incidence of perforation than diagnostic ones [4,6].

Abdominal compartment syndrome (ACS) refers to organ dysfunction that may occur as a result of increased intra-abdominal pressure (IAP)[7,8]. It may be classified as acute primary, secondary or recurrent according to its cause and duration [9,10].

We report a life threatening case of primary acute abdominal compartment syndrome (AACS) resulting from iatrogenic colonic perforation during a diagnostic colonoscopy.

## Case presentation

We report the case of a 60-year-old Moroccan man admitted for the surgical management of a high rectal adenocarcinoma. He had no past history of cardiovascular or pulmonary disease, with no recent surgery, and was classified according to the American Society of Anesthesiology (ASA) as ASA II. He was scheduled to undergo a second colonoscopy by an expert endoscopist to eliminate a second colonic tumor before an anterior resection. Preoperative anesthesia investigations, including all blood tests, a chest X-ray and an electrocardiogram, were unremarkable.

On the day of the colonoscopy, our patient's blood pressure was 120/90 mmHg, his heart rate was 75 beats/minute, his respiratory rate was 12 breaths/minute, his body temperature was 37°C and he had a blood oxygen saturation level of 99% at ambient air.

Our patient had the procedure under conscious sedation with the presence of an anesthetist nurse. During the first five minutes of the procedure, the endoscopist reported some difficulties, but no signs of perforation, that caused a little discomfort and pain to our patient, which was managed with two 1 mg intravenous bolus injections of midazolam. This allowed our patient to remain awake and follow the endoscopist's instructions.

At that time his vital signs were unchanged except of a rise in his heart rate. At the end of the examination of his ascending colon, our patient developed progressive bradycardia. The endoscopist noticed a significant abdominal distension followed by respiratory distress that rapidly progressed to cardiac arrest. The procedure was immediately stopped and cardiopulmonary resuscitation begun. The reanimation team was called for intubation while closed chest cardiac massage was initiated by the endoscopic and surgical team.

Our patient was intubated and manually ventilated with 100% oxygen and epinephrine was administered. Despite these measures, the cyanosis worsened and his pulse was still not palpable.

**Figure 1:**
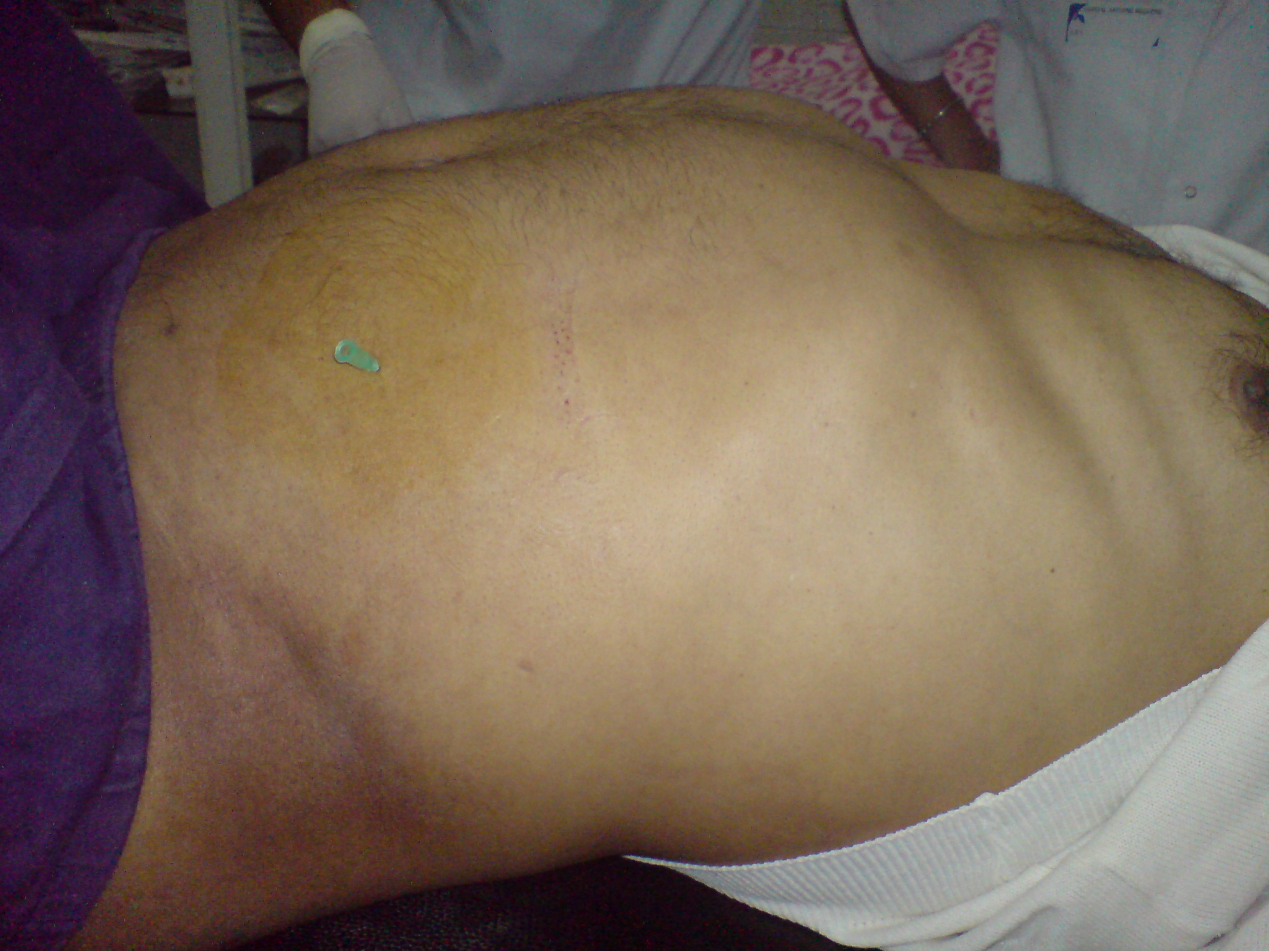
**Fine needle decompression as a salvage action to decompress acute abdominal compartment syndrome causing cardiac arrest with pulselessness**.

After five minutes, a 14-gauge needle was used to decompress the abdominal tension as a last procedure to avoid a salvage thoracotomy (Figure 1). His pulse was then detected, and his blood pressure started to rise, returning to the preoperative state. Our patient stabilized and was transferred to the intensive care unit.

A diagnosis of AACS due to iatrogenic colonic perforation was made and our patient managed accordingly. However, while he was in the intensive care unit, no intra-abdominal pressure measure was realized. Three hours later, our patient underwent a laparotomy and a sigmoid perforation was found just 3 cm upstream of the tumor. We decided to perform an anterior resection, including the perforation site in his sigmoid colon, with primary colorectal anastomosis protected by an ileostomy.

Our patient had no postoperative complications and was discharged on the tenth day. He returned after two months for the closure of the ileostomy and remains free of disease three years later.

## Discussion

We describe this rare case of colonic perforation leading to AACS with pulselessness and cardiac arrest that was quickly and efficiently managed by a puncture decompression.

Clinical presentations of these perforations depend on the size, site and mechanism of perforation, the underlying pathology, the degree of peritoneal contamination and the condition of the patients [1,3,5]. The most common immediate symptom is abdominal pain occurring during or after the procedure. However, a silent perforation, not detected by the endoscopist during the procedure, may lead him to increase air insufflation for a better visualization, provoking an air extralumenization in peritoneal cavity or retroperitoneum, leading to subcutaneous emphysema, pneumoscrotum, pneumopericardium or a pneumothorax and possibly causing tension pneumoperitoneum [11,12].

AACS is a complex pathologic state in which an increase in IAP (the pressure concealed within the abdominal cavity) results in physiologic derangements in several organ systems [9,13]. ACS is defined by the World Society of the Abdominal Compartment Syndrome as a sustained IAP over 20 mmHg associated with organ failure [10].

This resulting intra-abdominal hypertension (IAH) leads to both cardiologic and pulmonary symptoms [9,13]. The cardiologic symptoms are due to the combination of a decreased preload by the compression of the inferior vena cava and portal vein, and an increased afterload due to increased systemic vascular resistance by the IAH, leading to a decreased stroke volume and thus decreased cardiac output and cardiac arrest with pulselessness [14]. The respiratory symptoms are due to the upward displacement of the diaphragm with a resultant decreased total lung volume, residual lung volume and lung compliance [13,15].

The most common causes of increased IAP are abdominal surgery complicated by pre- or postoperative bleeding, acute abdominal trauma, retroperitoneal bleeding after aortic surgery and extended burns to the abdominal wall [9]. However, the frequency of the use of laparoscopic surgery has led some authors to add pneumoperitoneum as a potential new cause of this syndrome [13].

The unique and specific treatment of this exceptional acute state, when suspected, is abdominal decompression, performed by a decompressive abdominal laparotomy or an air puncture. Although the use of a 14-gauge needle seems to be so simple as to be inadequate, in our case it represented the salvage procedure after the failure of cardiopulmonary resuscitation. It permitted decompression of the IAH, restoring a normal pulse, blood pressure and respiratory rhythm.

This can be explained by the high compliance and elasticity of the abdominal wall, similar to the pericardium. The pericardial pressure to volume relationship is curvilinear [16]: the pericardium may become relatively distended on an initial increase in pericardial volume, but its compliance becomes extremely reduced after a certain degree of distention, so that a small increase in subsequent pericardial volume is accompanied by a significant rise in the pressure [16].

There is a prediction model described by Papavramidis *et al*., applied to IAH, that shows that there is a linear relationship between the volume introduced or extracted from the abdominal cavity and intra-abdominal pressure [17]. This has been further supported by the draining of ascetic and pancreatic fluid [9,15]. Otherwise, the removal of only a few cubic centimeters of air in AACS is sufficient to restore an efficient preload by decompressing the vena cava and restarting the heart activity [10].

The management of iatrogenic colonic perforation is still controversial, with two options-operative and non-operative management. However, given the high risk of mortality (up to 50%) and morbidity of these complications, many authors insist on quick surgical management [1].

The absence of any significant contamination (after colonic preparation for this procedure) makes it possible to perform an initial repair or, like in our case, radical surgery with a hemicolectomy and an immediate colorectal anastomosis.

To the best of our knowledge, only two similar cases have previously been reported. In the first case, the patient underwent conservative treatment to manage a colonic perforation resulting from a diagnostic colonoscopic examination of a recently known constructed mucus fistula [14]. In the second case, the patient underwent an urgent laparotomy to repair an intestinal perforation after failed endoscopic clamping [18].

## Conclusion

During a colonoscopy, the endoscopist and anesthesiologists should be aware of this rare complication that may occur due to asymptomatic colonic perforation, and keep in mind the salvage action of fine needle decompression, because failure to recognize and treat AACS is inevitably fatal.

## Abbreviations

AACS: acute abdominal compartment syndrome; ACS: abdominal compartment syndrome; ASA: American Society of Anesthesiologists; IAH: intra-abdominal hypertension; IAP: intra-abdominal pressure.

## Consent

Written informed consent was obtained from the patient for publication of this case report and any accompanying images. A copy of the written consent is available for review by the Editor-in-Chief of this journal.

## Competing interests

The authors declare that they have no competing interests.

## Authors' contributions

AS was the author in charge and wrote the manuscript. RM, LI and AB helped in writing the manuscript. HOEM was a major contributor in writing the manuscript. All authors read and approved the final manuscript.
